# The effects of ANC follow up on essential newborn care practices in east Africa: a systematic review and meta-analysis

**DOI:** 10.1038/s41598-021-91821-z

**Published:** 2021-06-09

**Authors:** Erkihun Tadesse Amsalu, Bereket Kefale, Amare Muche, Zinabu Fentaw, Reta Dewau, Muluken Genetu Chanie, Mequannent Sharew Melaku, Melaku Yalew, Mastewal Arefayine, Gedamnesh Bitew, Bezawit Adane, Wolde Melese Ayele, Yitayish Damtie, Metadel Adane, Tefera Chane Mekonnen

**Affiliations:** 1 Department of Epidemiology and Biostatistics, School of Public Health, College of Medicine Health Sciences, Wollo University, Dessie, Ethiopia; 2 Department of Reproductive and Family Health, School of Public Health, College of Medicine Health Sciences, Wollo University, Dessie, Ethiopia; 3 Department of Health Systems and Policy, School of Public Health, College of Medicine Health Sciences, Wollo University, Dessie, Ethiopia; 4 Department of Health Informatics, Institute of Public Health, College of Medicine and Health Sciences, University of Gondar, Gondar, Ethiopia; 5 Department of Environmental Health, College of Medicine and Health Sciences, Wollo University, Dessie, Ethiopia; 6 Department of Nutrition and Dietetics, School of Public Health, College of Medicine and Health Sciences, Wollo University, Dessie, Ethiopia

**Keywords:** Epidemiology, Health care, Medical research

## Abstract

In the situation of high maternal morbidity and mortality in Sub-Saharan Africa, less than 80% of pregnant women receive antenatal care services. To date, the overall effect of antenatal care (ANC) follow up on essential newborn practice have not been estimated in East Africa. Therefore, this study aims to identify the effect of ANC follow up on essential newborn care practice in East Africa. We reported this review according to the Preferred Reporting Items for Systematic Reviews and Meta-Analysis (PRISMA). We searched articles using PubMed, Cochrane library, African journal online (AJOL), and HINARI electronic databases as well as Google/Google scholar search engines. Heterogeneity and publication bias between studies were assessed using I^2^ test statistics and Egger’s significance test. Forest plots were used to present the findings. In this review, 27 studies containing 34,440 study participants were included. The pooled estimate of essential newborn care practice was 38% (95% CI 30.10–45.89) in the study area. Women who had one or more antenatal care follow up were about 3.71 times more likely practiced essential newborn care compared to women who had no ANC follow up [OR 3.71, 95% CI 2.35, 5.88]. Similarly, women who had four or more ANC follow up were 2.11 times more likely practiced essential newborn care compared to women who had less than four ANC follow up (OR 2.11, 95% CI 1.33, 3.35). Our study showed that the practice of ENBC was low in East Africa. Accordingly, those women who had more antenatal follow up were more likely practiced Essential newborn care. Thus, to improve the practice of essential newborn care more emphasis should be given on increasing antenatal care follow up of pregnant women in East Africa.

## Introduction

According to the World Health Organization (WHO) essential newborn care utilization is a strategic approach to improve the health of newborns through interventions rendered prior to conception, during pregnancy, during delivery and soon after birth and during the postnatal period^1^.

Globally, neonatal death accounts for about 44% of under-five mortality and Sub-Saharan Africa (SSA) has the highest rates of neonatal mortality accounting 38% of all neonatal deaths^2^.

In developing countries, neonatal mortality has remained resistant to change^3,4^. About three-fourths of deaths among new-born occur in the first week of life, and 25–40% occurs in the first 24 h^5,6^.

Most causes of neonatal death are preventable and related to cord care to decrease sepsis, temperature control by delaying first bath and initiation of early breastfeeding which has the additional benefit of controlling hypothermia^7–10^.

Essential Newborn Care (ENBC) utilization is one of the recommended strategies by World Health Organization (WHO) to reduce neonatal mortality and morbidity in both community and facility delivery^11^. ENBC practices include clean cord care, thermal care and initiating breast feeding immediately or within the first hour after birth^3,6,12,13^.

The Ethiopian government has paid special attention to the expansion of quality high impact neonatal interventions by establishing basic newborn care units in health centers and in hospital’s neonatal intensive care units^14,15^.

Most newborn deaths occur at home in low- income countries including east African regions due to a lack of access to health care, a limited number of trained health care personnel, and an overall weak health system in the regions^16^.

Hence for safe home based deliveries new strategies should be developed. Thus, essential newborn care practice should be designed for neonates who cannot be managed at home in ensuring proper referral. Further, in domestic settings it would help as an appropriate means of the newborns care^17^.

In developing countries there is low level of adherence to essential new-born care practice despite this recommendation. Accordingly, reports from east African countries demonstrated the low level of essential new-born care practice^16,18–21^. For example in Uganda early bathing and spread over materials on cord stump is a custom^21–23^. Similarly, studies done in different parts of Ethiopia also demonstrated the low level of utilization of essential new-born care^6,24–28^.

In east Africa region the period after delivery is frequently noticeable by traditional practices. Bathing the baby immediately following delivery, applying diverse matters on the umbilical cord and giving various pre-lacteal feeds for the neonates were most important cultural performs hamper the health and survival of the newborn. Even if understanding these cultural practices is an essential part of confirming effective and timely newborn care; there is inadequate documented evidence in east Africa where sub-optimal newborn care practices has been widely described ^3,29,30^.

Antenatal period (ANC) is one of the instruments to obtain reduction in neonatal mortality by counseling pregnant women to have access for essential newborn care practices preparing them to care for their newborn when they visit health facilities and during home visits made by community health workers^3,31^.

Various studies have assessed factors affecting essential newborn care utilization in East Africa^6,24–28^. But none of them identified the pooled effect of ANC on essential new born care use in East Africa. Therefore, this meta-analysis summarizes the effect of ANC on essential newborn care practice among women in East Africa systematically and quantitatively. Thus, the findings will help to determine how much of an impact have antenatal care follow up on essential newborn care practice in East Africa. In addition, this study provides comprehensive information for policy makers and program managers to design strategies that increase the utilization of essential newborn care.

## Methods

### Searching strategies

This systematic review has been prepared according to the Preferred Reporting Items for Systematic Review and Meta-Analysis (PRISMA) guideline^32^. The systematic review was registered on the PROSPERO prospective register of systematic reviews (registration number: CRD42020207894).

We searched articles using PubMed, Cochrane library, African journal online (AJOL), and HINARI electronic databases as well as Google/Google scholar search engines.

A medical subject head (MeSH) and keyword items was used in separate and combination using Boolean operator: “OR”, “AND” or “NOT” in order to identify relevant articles. Thus, searches was conducted using Keywords/search terms like “Prevalence”, “proportion”, “magnitude”, “essential”, “essential newborn care” “newborn”, “neonatal care”, “newborn care”, “utilization”, “services”, “practices”, “Antenatal care”, “prenatal care”, “maternal health care”, “delivery care”, “East Africa”.

### Inclusion criteria’s

Study design and period: all observational studies conducted in East Africa published until September, 2020 were included in this review and meta- analysis.

Participants: women who delivered either in the health facility or at the home.

Setting: All community-based and facility-based studies reported ENBC utilization and ANC follow up.

Language: Articles published in English language.

Exposure: ANC follow up.

Outcome: Essential newborn care (ENBC) utilization/practice.

We excluded Primary studies inaccessible for full-text article after 2 times author request. In addition, those articles published in non-English languages were also excluded from this review and meta-analysis.

### Data extraction and synthesis

A standardized data extraction format was prepared in the form of Microsoft Excel.

The data extraction format includes author, year of publication, study country, year of publication, study period, study setting, study design, sample size, number of subjects with outcome, ANC follow up, and essential newborn care practice.

Three reviewers (ETA, MY, BK) independently extracted the data and any disagreements between the reviewers were solved through consensus. For incomplete data, we excluded the study after attempts to contact the corresponding author through email.

The quality of the studies was assessed using Joana Briggs Institute (JBI) adapted for observational studies by 2 Authors (MA, WM) before analysis and any discrepancy was resolved by discussion and consensus^33,34^.

### Heterogeneity and publication bias

We identified the heterogeneity between the studies using I^2^ statistics and P values^35^. A forest plot was used to detect the presence of heterogeneity. Furthermore, subgroup analysis and meta-regression was used to identify the possible source of heterogeneity.

To check publication bias, both objective and subjective (funnel plot) methods were used. The presence of publication bias was checked using subjective method (funnel plot) symmetry. In addition, the statistical significance of publication bias was assessed using objective method s Egger’s and Beggar’s test (P < 0.05)^36,37^.

### Statistical analysis

After extracting the data, we imported into STATA Version 14.0 statistical software for further analysis. The analysis to identify the effect of ANC follow up on essential newborn care service use was categorized into two parts. The first analysis was to identify the effect of one or more ANC visits on essential newborn care service use and the second was an analysis of the effect of four or more ANC visits on essential newborn care service use. A random effects meta-analysis model was employed to estimate the DerSimonian and Laird’s pooled effect because of high levels of heterogeneity^38^. The results were presented in tables and forest plot.

## Results

### Study selection and screening

**Figure 1:**
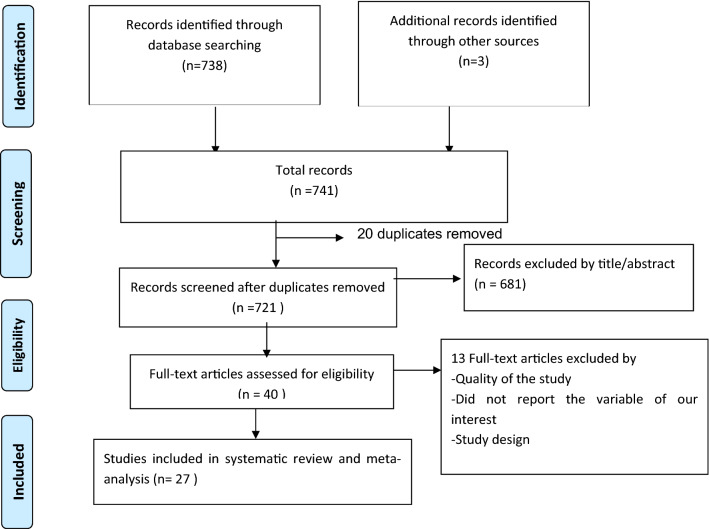
PRISMA flow diagram of studies included to estimate the effect of ANC follow up on ENBC practice in East Africa in the systematic review and meta-analysis.

As described in Fig. 1 a total of 741 records were retrieved through database searching. Of this 20 articles were removed due to duplication and about 681 articles were removed after reviewing their title and abstracts. About 40 articles were selected for full text review. From these, 5 studies were excluded for not reporting the outcome variable, 5 studies by the study design, and 3 studies by quality of the study. Finally, 27 articles met the eligibility criteria included in the final Meta-analysis (Fig. 1, Table 1).

**Table 1:** Characteristics of included studies to explore the effect of ANC follow up on Essential newborn care practice in East Africa.

Author (year)	Study area	Sample size	Study design	Study period	JBI score (%)
Chichiabellu et al. (2018)	Ethiopia	450	CB cross-sectional	2016	80
Gebrehiwot et al. (2020)	Ethiopia	634	CB cross-sectional	2018	80
Mersha et al. (2018)	Ethiopia	630	CB cross-sectional	2017	70
Etafa et al. (2020)	Ethiopia	417	FB cross-sectional	2017	80
Komakech et al. (2020)	Uganda	561	CB cross-sectional	2016	60
Mamush et al. (2020)	Ethiopia	495	CB cross-sectional	2019	70
Tafere et al. (2018)	Ethiopia	970	FB follow up	2016	80
Amsalu et al. (2019)	Somalia	332	FB cross-sectional	2016	70
Workinesh et al. (2019)	Ethiopia	576	FB cross-sectional	2018	60
Berhea et al. (2018)	Ethiopia	456	CB cross-sectional	2016	80
Yimam et al. (2015)	Ethiopia	539	CB cross-sectional	2013	60
Kasaye et al. (2020)	Ethiopia	390	FB cross-sectional	2017	70
Semanew et al. (2019)	Ethiopia	418	FB cross-sectional	2018	80
Waiswa et al. (2010)	Uganda	414	CB cross-sectional	2007	70
Miriam et al. (2020)	Rwanda	192	CB cross-sectional	2019	80
Teshome et al. (2015)	Ethiopia	570	CB cross-sectional	2013	80
Misgna et al. (2016)	Ethiopia	296	CB cross-sectional	2014	80
Kebede (2019)	Ethiopia	414	FB cross-sectional	2016	80
Rosales et al. (2014)	South Sudan	511	CB cross-sectional	2013	70
Alemu (2020)	Ethiopia	834	CB cross-sectional	2018	60
Penfold et al. (2010)	Tanzania	22,243	CB Retrospective cross-sectional	2007	60
Kayom et al. (2015)	Uganda	338	CB cross-sectional	2012	70
Berha et al. (2017)	Ethiopia	215	FB cross-sectional	2016	70
Gebremedhin et al. (2020)	Ethiopia	371	CB cross-sectional	2017	60
Lucia et al. (2017)	Kenya	380	FB cross-sectional	2013	60
Meseka et al. (2017)	South Sudan	384	FB cross-sectional	2015	60
Kabwijamu et al. (2016)	Uganda	410	CB cross-sectional	2014	80

### Description of studies

In this review, a total of 34,440 study participants from 27 studies were included. The studies were conducted from 2007 to 2019 and published from 2010 to 2020. Regarding study country, most of the studies (17) were conducted in Ethiopia. The sample size of the included studies ranged from 192 to 22,243. In terms of study design, majority of the studies (26) were cross-sectional. From a total of 27 articles included in this review, about 17 of them were community-based and the remaining 10 were institutional-based studies (Table 1).

The quality of the studies was assessed using Joana Briggs Institute (JBI) adapted for observational studies by independent evaluators before analysis. We included those studies that fitted to 50% and above the quality assessment checklist. Hence, the results of quality assessment ranged from 60 to 80% and all studies were fit for analysis (Table 1).

### Meta-analysis

**Figure 2:**
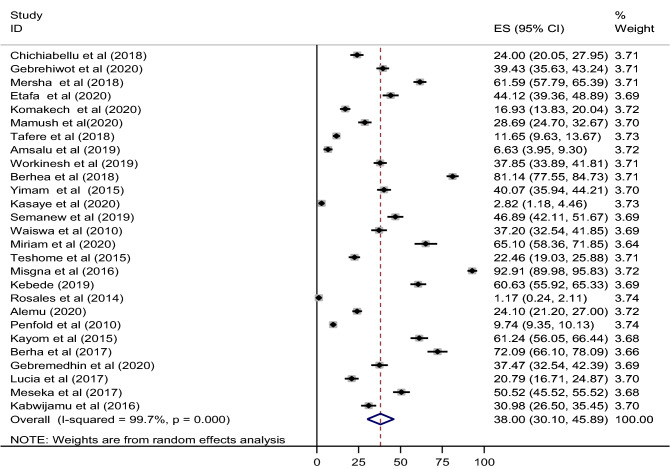
Forest plot of the pooled estimate of essential newborn care practice in east Africa.

In this review and meta-analysis about 27 studies were included to estimate the pooled estimate of essential newborn care practice among women in East Africa. The level of essential newborn care practice was ranged 1.17–92.91% from the included studies (Fig. 2). As illustrated in Fig. 2, the overall pooled estimate of essential newborn care practice was 38% (95% CI 30.10–45.89). In this study, I^2^ statistic was detected considerable and significant heterogeneity between studies (I^2^ = 99.7, P value < 0.0001). Therefore, a random effect model was used to estimate the overall pooled prevalence of essential newborn care practice in East Africa.

We conducted meta-regression using different factors to identify the possible source of heterogeneity between studies. But, there was no statistical significance variation from the meta-regression result. In addition, subgroup analysis was conducted to explore the source of heterogeneity in Essential newborn care practice using different factors included in the study. But, no significant change was seen as compared with the main meta-analysis. Hence, the heterogeneity might be explained by other covariates which are not included in this study.

Furthermore, a sensitivity analysis was done to identify influential studies. According to the analysis, all of the studies were included in the final analysis since no influential studies were detected.

### Publication bias

**Figure 3:**
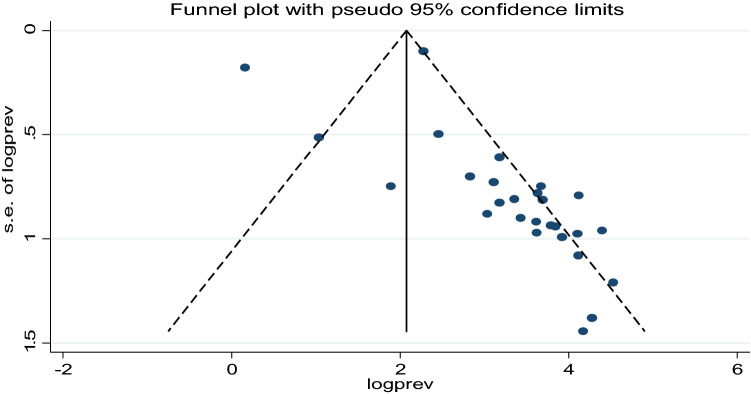
Funnel plot of the meta-analysis containing 27 studies.

We have checked publication bias of the included studies using Funnel plot and egger tests. Accordingly, Egger’s test showed no statistically significant publication bias (P = 0.64). As depicted in Fig. 3 the funnel plot has also indicated the absence of publication bias since studies represented by dots were asymmetrical (Fig. 3).

### Effect of antenatal care follow up on essential newborn care practice

**Figure 4:**
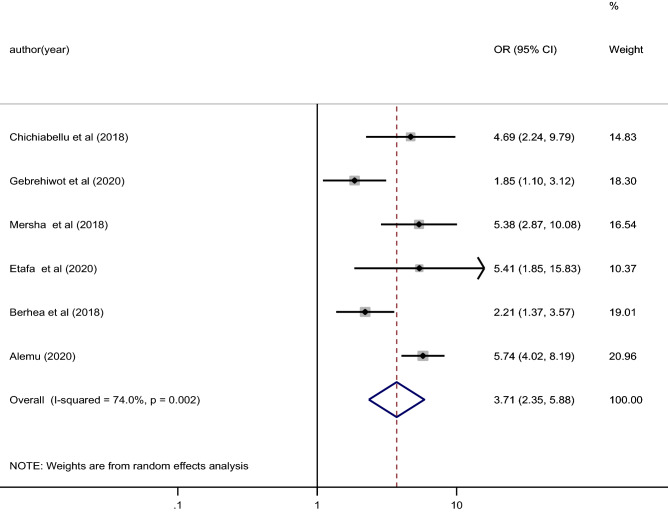
The pooled effect of ANC visit on Essential newborn practice among women in East Africa.

We included a total of 6 cross- sectional studies to estimate the effect of ANC follow up on essential newborn care practice in east Africa. The participants of the studies ranged from 417 to 834 (Table 2). The included studies showed significant heterogeneity and detected using I^2^ statistic (I^2^ = 74.0%, P value = 0.002) (Fig. 4). Therefore, a random effect model was used to estimate the overall pooled effect of ANC follow up on essential newborn care in the study area.

**Table 2:** Characteristics of studies included to study the effect of ANC visit on essential newborn care practice in East Africa.

Author (year)	Study area	Study design	Sample size	Study period	ANC visit	ENBCP	
						Yes	No
Chichiabellu et al. (2018)	Ethiopia	CB cross-sectional	450	2016	Yes	18	14
					No	90	328
Semanew et al. (2019)	Ethiopia	FB cross-sectional	418	2018	Yes	238	321
					No	22	55
Mersha et al. (2018)	Ethiopia	CB cross-sectional	630	2017	Yes	230	303
					No	12	85
Etafa et al. (2020)	Ethiopia	FB cross-sectional	417	2017	Yes	180	208
					No	4	25
Berhea et al. (2018)	Ethiopia	CB cross-sectional	456	2016	Yes	262	45
					No	108	41
Alemu (2020)	Ethiopia	CB cross-sectional	834	2018	Yes	543	103
					No	90	98

As described in Fig. 4 the random effect model analysis showed, women who had one or more antenatal care follow up were 3.71 times more likely practiced essential newborn care compared to mothers who had no ANC visit [OR 3.71, 95% CI 2.35, 5.88] (Fig. 4).

In order to identify the effect of four or more ANC follow up on essential newborn care practice about 4 additional studies with 2140 participants were included. The studies were conducted from 2012 to 2018 and published from 2015 to 2019 (Table 3).

**Table 3:** Characteristics of studies included to identify the effect of number of ANC visits on essential newborn care practice in East Africa.

Author (year)	Study area	Study design	Sample size	Study period	ANC visit	ENBCP	
						Yes	No
Tafere et al. (2018)	Ethiopia	FB follow up	450	2016	≥ 4	56	171
					< 4	57	539
Kebede (2019)	Ethiopia	FB cross-sectional	414	2016	≥ 4	7	3
					< 4	191	206
Mersha et al. (2018)	Ethiopia	CB cross-sectional	630	2017	≥ 4	160	73
					< 4	82	86
Kayom et al. (2015)	Uganda	CB cross-sectional	338	2012	≥ 4	107	60
					< 4	100	69

**Figure 5:**
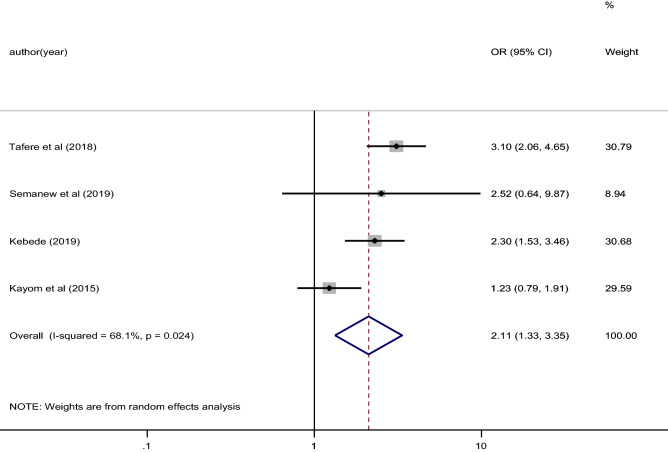
The pooled effect of four or more ANC follow up on essential newborn care practice in East Africa.

Significant heterogeneity was observed between studies and detected using I^2^ statistic (I^2^ = 68.1, P = 0.024) (Fig. 5). Thus, a random effect model was used to estimate the pooled effect of four and more ANC follow up on essential newborn care practice in the study area (Table 3).

The pooled effect of 4 studies revealed having four or more ANC follow up was significantly associated with essential newborn care practice in East Africa. Figure 5 illustrated women who had four or more ANC follow up were 2.11 times more likely practiced essential newborn care compared to women who had less than four ANC follow up (OR 2.11, 95% CI 1.33, 3.35).

## Discussion

Antenatal care has been used as a strategy to reduce maternal and newborn morbidities and mortalities. In developing countries, different strategies have been implemented to improve the effectiveness of Antenatal care. Nowadays, most developing regions including East African regions used the focused ANC approach which was developed by WHO^39,40^.

Thus, this systematic review and meta-analysis aims to estimate the pooled prevalence of newborn care practice and identify the effect of ANC follow up on essential newborn care practice among post-partum women in East Africa.

This review study estimated that the level of Essential newborn care practice among postpartum women in East Africa was 38%. This finding was lower as compared to a meta-analysis done in Ethiopia about 48.77%^7^, Nepal about 70.7%^41^, and India about 66.70%^42^. The possible justification for the discrepancy may be due to differences in socio-economic, differences in socio-cultural aspects, differences in study setting, and differences in sample size. In addition, the discrepancy may be due to the variation in maternal health services coverage across countries based on increased awareness and information about ENBC utilization.

This study revealed that women who attended antenatal care were more likely practiced essential newborn care in the study regions.

Concordant to this result there was a report from low income country^43^ and Northern Ghana^44^. This could be related that mothers who visited Antenatal care follow up had an opportunity of obtaining health workers information about the importance of newborn care practice through attending health facilities. Further, Antenatal care has a positive association with essential newborn practices including clean cord care and thermal care^44^. Furthermore, during immediate ANC visits they told from health professional about essential newborn care practice that helps to provide appropriate and timely periodic advice consult which would in turn increases the utilization newborn care^45^.

This review and meta-analysis had using large sample size, which meant that it could detect the effect of ANC on essential new born care practice. However, the study does not address other factors that influence essential newborn care practice.

This research is important for understanding the effect of ANC follow up on essential newborn care practice in East Africa. The finding is also relevant for policymakers to establish criteria for improving the quality of maternal and newborn care in East Africa.

## Conclusion

This meta-analysis revealed that Essential newborn care practice among women in East Africa was low. Women who had one or more antenatal care follow up were more likely practiced essential newborn care in East Africa. Thus, women who attend four or more Antenatal care visits were more practiced essential newborn as compared to those who have less than four follow ups in the study area. Therefore, the governments and concerned bodies should give special attention to increase Antenatal care follow up of women to improve essential newborn care use.

## Abbreviations

ANC: Antenatal care
ENBC: Essential newborn care
WHO: World Health Organization
